# Immense Tumor of Maxillary Sinus with Exophthalmos—A Rare Underlying Cause

**DOI:** 10.3390/hematolrep14040044

**Published:** 2022-10-10

**Authors:** Olga Lesniewska-Skowerska, Joanna Symela-Kaspera, Lucyna Klimczak-Gołąb, Wojciech Smolka, Jaroslaw Markowski

**Affiliations:** Department of Laryngology, Faculty of Medical Sciences in Katowice, Medical University of Silesia, 40-055 Katowice, Poland

**Keywords:** lympoma, sinus, maxillary, tumor

## Abstract

Sinus tumors are arduous to diagnose due to often prolonging asymptomatic course until the infiltration of the adjacent structures occurs. Therefore, patients are diagnosed with advanced-stage disease, which negatively affects the treatment outcomes. A 60-year-old male was referred to our ward from an outpatient clinic. The patient presented with double vision, right-side lacrimation, and exophthalmos. He also reported significant weight loss: 15 kg in 2 months. Physical examination revealed achiness and edema of the right temporal area, and subconjunctival hemorrhage of the right eye, and surprisingly no anosmia, no nasal obstruction, and no head/neck lymphadenopathy were present. The histopathology examination identified diffuse large B-cell lymphoma (CD20+ CD3- p63- bcl-2+ CD23-/+ bcl-6+ CD 10- MUM1+ Tdt- CD38- cyclin D1- CD30- c-myc+). The patient was qualified for R-CHOP immunochemotherapy (rituximab, cyclophosphamide, and hydroxydaunorubicin hydrochloride), which was well tolerated. After 3 months of treatment, all of the symptoms reversed and a whole-body PET scan showed no abnormal metabolic activity.

## 1. Introduction

Sinus tumors are arduous to diagnose due to often prolonging asymptomatic course until the infiltration of the adjacent structures occurs. Therefore, patients are diagnosed with advanced-stage disease, which negatively affects the treatment outcomes [1]. 

Diffuse large B-cell lymphoma (DLBCL) is characterized by significant heterogeneity in clinical symptoms, histological morphology, and prognosis. DLBCL is the most frequent type of non-Hodgkin lymphoma and occurs mainly in elderly (older than 60 years of age) men. There are two types of DLBCL: nodal and extranodal based on primary sites of origin. DLBCL has multiple locations. The most common are the gastrointestinal tract and the head-and-neck region. The maxillary sinus is rarely affected by DLBCL. 

## 2. Clinical Data

A 60-years-old male was referred to our ward from an outpatient clinic. The patient presented with double vision, right-side lacrimation, and exophthalmos. He also reported significant weight loss: 15 kg in 2 months. Physical examination revealed achiness and edema of the right temporal area, and subconjunctival hemorrhage of the right eye, and surprisingly no anosmia, nasal obstruction, and head/neck lymphadenopathy were present. The International Prognostic Index (IPI) was calculated at 2 points, which represents low–intermediate risk.

**Figure 1:**
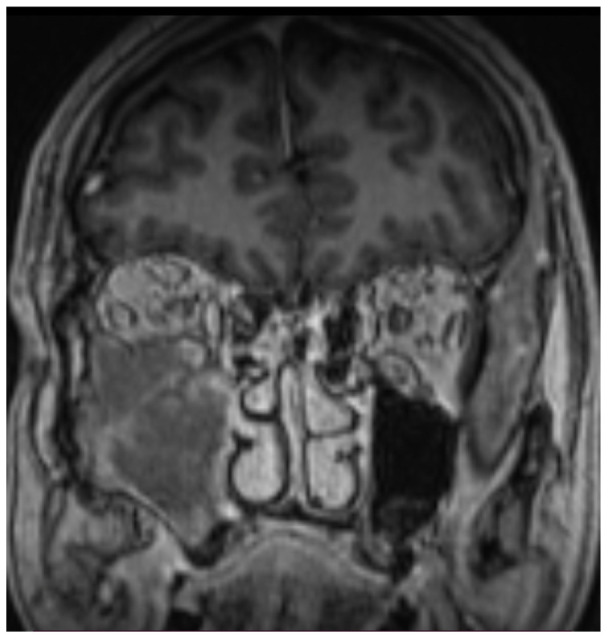
Head MR scan: large tumor evolving from maxillary sinus.

**Figure 2:**
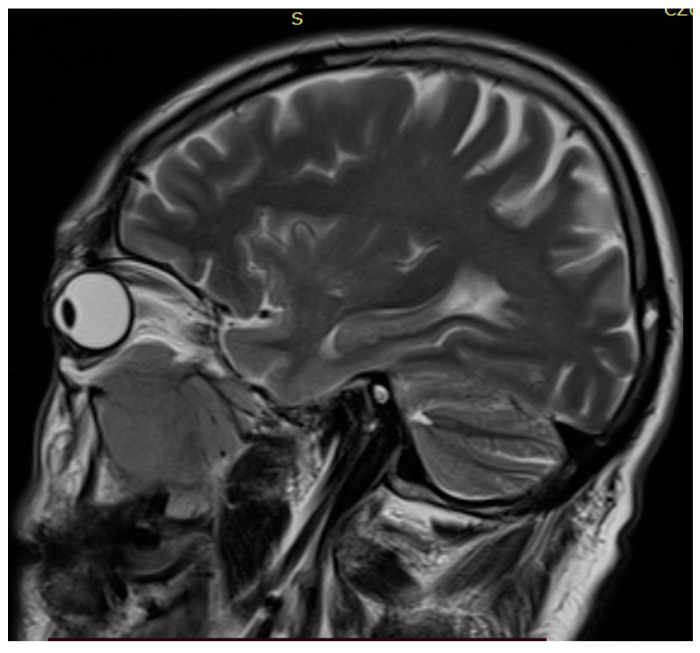
Head MR scan: large tumor evolving from maxillary sinus.

**Figure 3:**
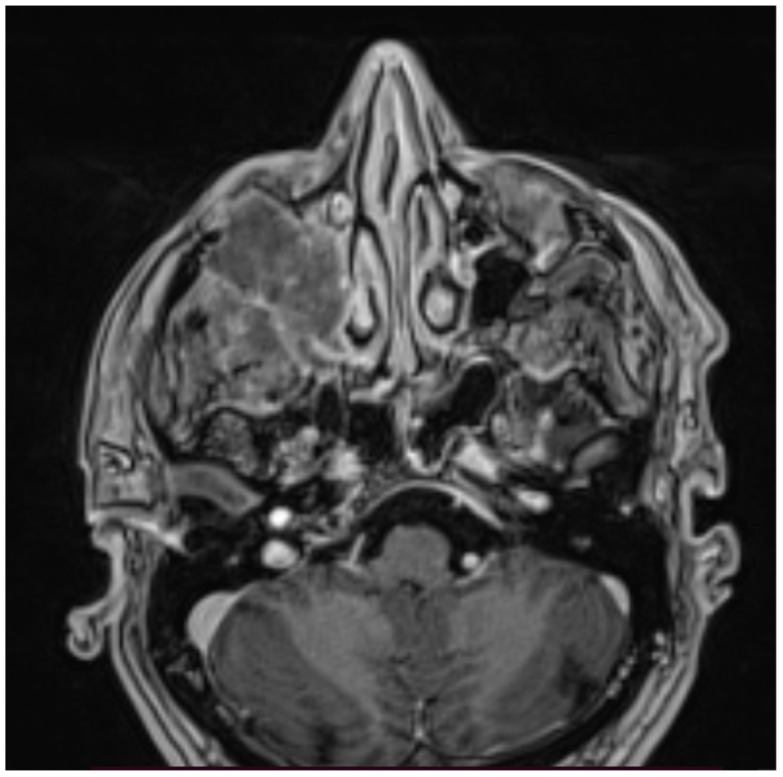
Head MR scan: large tumor evolving from maxillary sinus.

The head MR scan (T1 and T2 sequences) showed a large tissue mass (43 × 39 × 54 mm) penetrating the temporal and sub-temporal fossa, infiltrating the temporal muscle and the area of the right orbit, including the lower rectus muscle and infraorbital fat tissue (Figure 1, Figure 2 and Figure 3).

**Figure 4:**
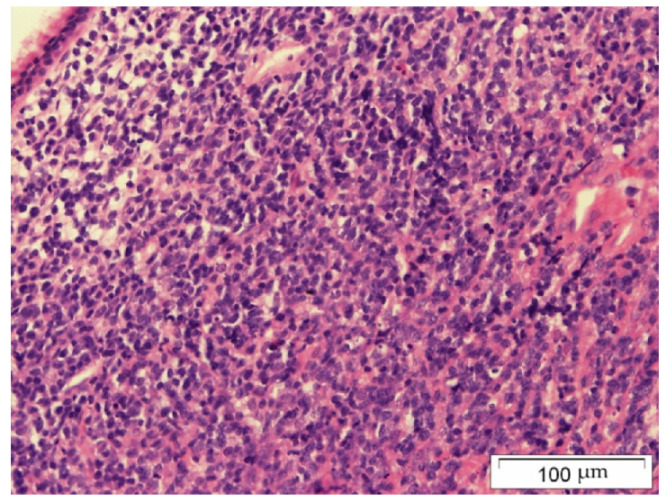
Tumor biopsy: HE staining.

**Figure 5:**
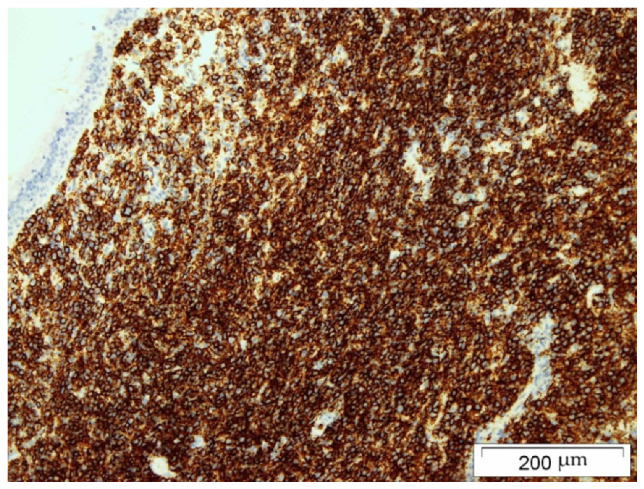
Tumor biopsy: CD20 staining.

**Figure 6:**
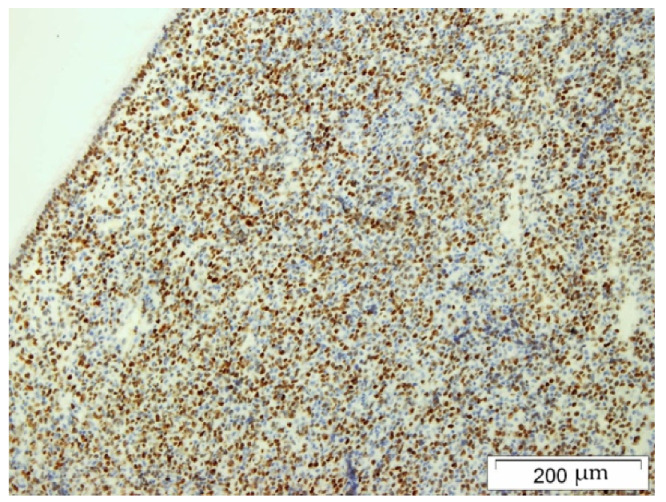
Tumor biopsy: bcl-6 staining.

**Figure 7:**
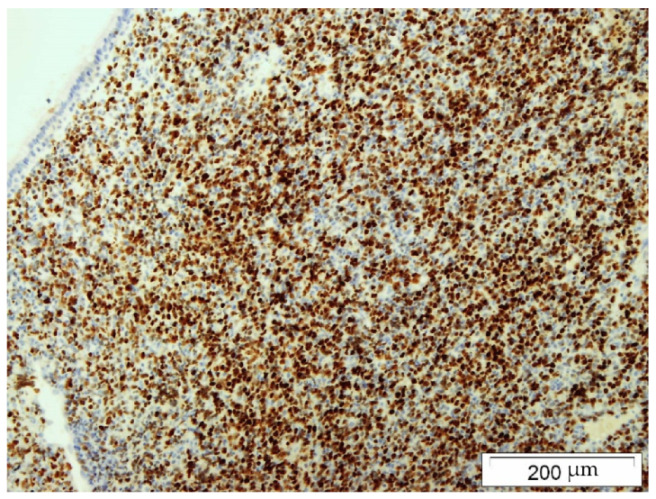
Tumor biopsy: MUM-1 staining.

The biopsy material was collected during Caldwell–Luc surgery in general anesthesia. The histopathology examination using the Hans algorithm identified diffuse large B-cell lymphoma (CD20+ CD3- p63- bcl-2+ CD23-/+ bcl-6+ CD 10- MUM-1+ Tdt- CD38- cyclin D1- CD30- c-myc+); Figure 4: tumor biopsy, HE staining; Figure 5: tumor biopsy, CD20 staining; Figure 6: tumor biopsy, bcl-6 staining; and Figure 7: tumor biopsy, MUM-1 staining). The CT scans with the contrast of the head, abdomen, and chest ruled out lymphoma lesions and confirmed the maxillary sinus as the origin of the tumor.

The patient was qualified for six cycles of R-CHOP immunochemotherapy (rituximab, cyclophosphamide, and hydroxydaunorubicin hydrochloride), which was well tolerated. The patient was not qualified for radiotherapy.

After 3 months of treatment, all of the symptoms reversed and a whole-body PET scan preformed at 3 and 9 months showed no abnormal metabolic activity.

## 3. Discussion

Lymphomas are rarely associated with sinus tumors and the clinical and radiographical manifestation is undifferentiable from squamous cell carcinoma or an odontogenic tumor, cyst, or infection. A tumor biopsy is crucial for the diagnosis. The Hans algorithm (analyzing CD-10, bcl-6, and MUM-1 protein expression) is widely utilized to categorize DLBCL into the germinal center B-cell (GCB) and non-GCB subtypes in order to select the most effective treatment and predict prognosis [2].

A recently published and also the largest (over eight thousand patients) meta-analysis of lymphomas in nasal and oral cavities by Jiang and Yan [3] reports that:

- only 18.2% of all head and neck lymphomas are located in nasal cavities or sinuses;
- the most common (19.2%) form of treatment is immunochemotherapy;
- they are localized (51.5%) and the 5-year survival is 68.1%.

Those data are in line with the largest single-site nasopharyngeal lymphoma analysis by Hsueh et al. [4]. The presented patient had also a similar clinical profile: male, 60 years old, localized tumor, and good response to immunochemotherapy.

Our patient presented symptoms at an advanced stage of disease; in our opinion, earlier diagnosis, in this case, could have been possible only by chance. According to Toda et al., the prognosis of DLBCL with nasal or paranasal localization is good despite the advanced stage at the time of diagnosis [5].

In our opinion, lymphoma should always be taken into consideration when a patient with a nasopharyngeal mass is being diagnosed.
